# Unmasking dermatofibrosarcoma protuberans: Case report of an atypical presentation complicated by post-surgical excision

**DOI:** 10.1016/j.ijscr.2020.03.020

**Published:** 2020-04-03

**Authors:** YaQun Zhou (Arlene), Justin Chin, Millard D. Strutin, Christine M. Lomiguen

**Affiliations:** a Department of Primary Care, Touro College of Osteopathic Medicine, New York, NY, United States; b Department of Surgery, Saint Clare’s Denville Hospital, Denville, NJ, United States; c Department of Family Medicine, Lifelong Medical Care, Richmond, CA, United States; d Department of Pathology, Lake Erie College of Osteopathic Medicine, Erie, PA, United States

**Keywords:** Dermatofibrosarcoma protuberans, DFSP, Soft tissue tumor, Lipoma, CD34, Case report

## Abstract

**Introduction:**

Dermatofibrosarcoma Protuberans (DFSP) is a rare type of malignant tumor of the cutaneous soft tissue that typically involves the dermis and surrounding structures. The typical clinical presentation involves an asymptomatic, slowly enlarging, indurated plaque, usually found on the trunk.

**Presentation of case:**

Here we present an atypical presentation of DFSP in a patient with a recurrent mass that was initially diagnosed as a lipoma, with supporting literature and clinical considerations for post-surgical management

**Discussion:**

In its earlier stages, DFSP can mimic other benign or malignant dermatological pathologies, which can complicate diagnosis purely via history and physical exam. Atypical features such as prior surgical management, morphology, and location can complicate the clinical diagnosis of DFSP. Immunohistochemistry is often required for definitive diagnosis as DFSP is CD34+ and is histologically described as spindle-cells in whorled or storiform pattern, with uniform, small elongated cells.

**Conclusion:**

DFSP is a rare tumor of the skin and subcutaneous tissue. Though it commonly presents with induration, skin discoloration, and telangiectasias, it is important to keep DFSP as a differential in new masses found on previous surgical sites, especially if the previous mass had a possibility of recurrence.

## Highlights

• Dermatofibrosarcoma protuberans (DFSP) is a rare tumor that behaves in a benign manner during its early stages and has a potential for metastasis.
• DFSP can occur under previous surgical scars which can mask its development from lack of common tumor presentations.
• Wide margin excision should be considered for removal of tumor to ensure clean margins in cases where DFSP is suspected.

## 1 Introduction

Dermatofibrosarcoma protuberans (DFSP) is a rare malignant tumor of cutaneous soft tissue, with a prevalence of 0.8–4.5 cases per 1 million per year in the United States [1,2]. As the name suggests, the tumor often involves the dermis and soft tissues, with pedunculation and spread in advanced stages [3]. The typical clinical presentation involves a slowly enlarging and indurated plaque, usually found on the truncal region of the body [4]. Largely asymptomatic, overlaying skin can show telangiectasia, sclerodermiform changes, and reddish-brown discoloration [1]. Earlier lesions can have a varied appearance, ranging from red and blue discoloration at the margins to rarely as a non-raised, cutaneous nodule [5]. Ulcerations and bleeding can accompany more aggressive types and in later stage of disease, with increased size and amount of telangiectasias [6,7]. Clinical diagnosis is complicated as many dermatological pathologies can present similarly on initial observation, resulting in insufficient treatment and excision [8]. High clinical suspicion and surgical intervention are critical to achieving a good prognosis [1,2].

Here we present an atypical case of DFSP in a patient with a recurrent mass that was initially diagnosed as a lipoma, with supporting literature and clinical considerations for post-surgical management. This case was conducted at St. Clare’s Denville Hospital, a New Jersey Health System community hospital in the department of surgery and work has been reported in line with the SCARE criteria [9].

## 2 Case presentation

**Fig. 1:**
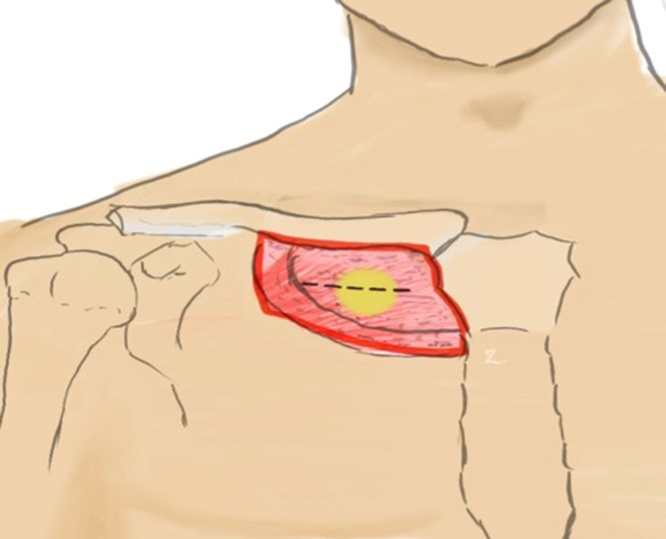
Illustrated diagram of mass location. Original illustration by YaQun Zhou (2020).

A 68-year-old Caucasian male presented for surgical evaluation with a right mid-infraclavicular chest mass from referral of his primary care physician (Fig. 1). Three years prior, the patient presented with a mass at the same location, subsequently diagnosed as a lipoma and locally excised with no surgical complications. At the time of the previous surgery, it was incidentally discovered and diagnosed that the patient also had diffuse B cell lymphoma, complete remission status post chemotherapy. Family, psychosocial, drug, and other surgical history were noncontributory. Over the past three years, the mass increased in size, but did not have any discoloration, discharge, or pain; however, due to the patient’s past medical history and immunocompromised state, the patient expressed concern and presented for elective excision of the mass.

On physical exam, a mass was palpated at the midclavicular line of the right upper chest, within the right first intercostal space. The mass was directly under the scar from previous excision, with no skin discoloration, telangiectasia, or visible raised nodules. The patient denied any pain, tenderness, or discharge associated with the new mass. Radiological testing was performed to determine the exact location and depth of invasion. CT chest with and without contrast revealed an ill-defined, cutaneous mass, while MRI chest was nondiagnostic.

Surgical excision was performed under local anesthesia, with a wide, elliptical incision to the level of the underlying bony structures to ensure clean margins. Excised specimen measured 5.5 cm × 3.5 cm × 2 cm with surrounding 4 cm × 1.6 cm × 0.4 cm of skin attached. The resulting wound was closed using interrupted 4–0 plain gut sutures for the full thickness, with 3–0 Vicryl subdermal and 4–0 Monocryl subcuticular reinforcing sutures.

**Fig. 2:**
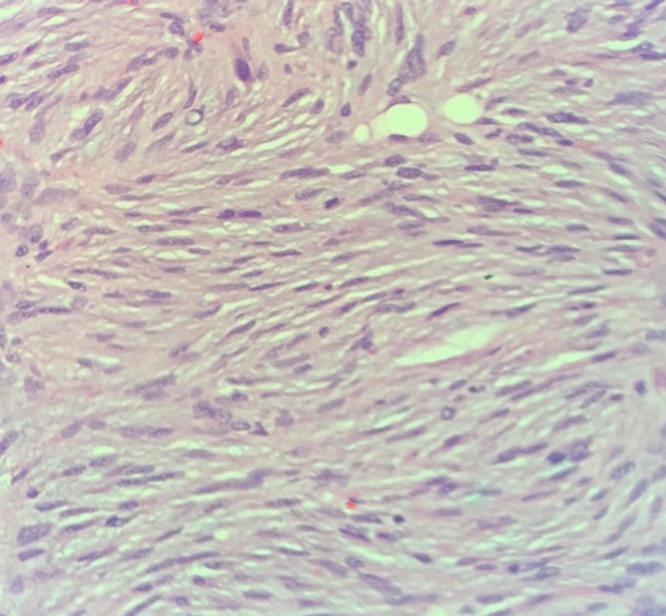
Hematoxylin and eosin stain of patient sample showing characteristic storiform array with mitotic figures.

**Fig. 3:**
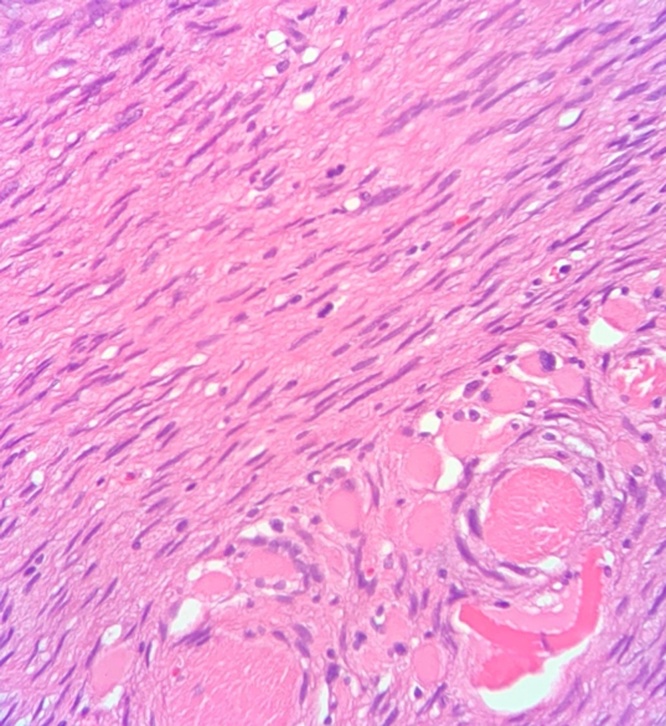
Hematoxylin and eosin stain of patient sample showing infiltration into subcutaneous fat.

**Fig. 4:**
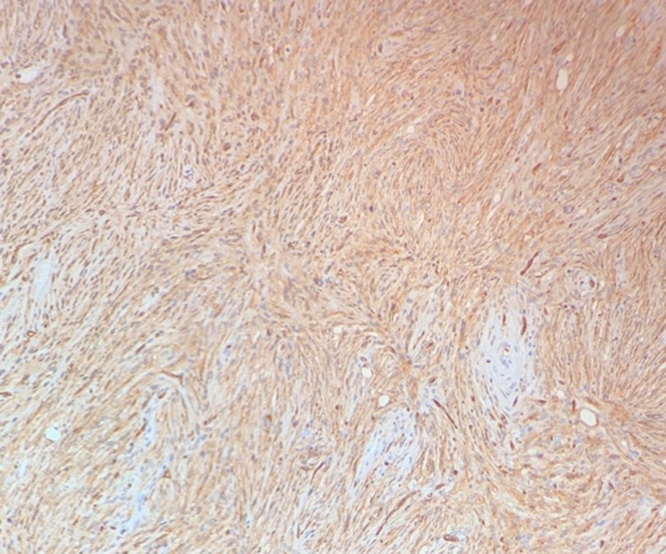
Immunohistochemistry stain of patient sample showing positive reaction for CD34 markers.

The specimen was sent to pathology for cryosectioning; however, due to increase in cellularity at the posterior margin, permanent fixation was performed. Within the submitted specimen, a subcutaneous firm nodule measuring 1.6 cm × 1 cm × 0.7 cm was located in the superior aspect with a 0.1 cm surrounding margin. Microscopic examination with hemolysin and eosin staining showed whorled myoid with spindled nuclei in storiform array throughout the dermis and extending into the subcutaneous fat with widening fibrous septa (Fig. 2, Fig. 3). The specimen was also composed of uniform, small elongated cells and scant cytoplasm with frequent mitotic figures and giant cells. The sample stained positively for CD34 and ultimately diagnosed as DFSP (Fig. 4).

As a result of the close margin excision, the patient was referred for radiation therapy with imatinib for recurrence prevention. Upon the last two follow-ups status post imatinib treatment, there have been no signs of recurrence.

## 3 Discussion

DFSP is invasive to surrounding tissues; the irregular, tentacle-like projections with septations and similarity to normal fibrous tracts of the tumor lends to difficulty in determining its borders, and consequently, its surgical margins for excision [3,6,10]. Variations of DFSP are distinguished by histological presentation and are commonly described histologically as benign-appearing spindle-cells, arranged in whorled or storiform pattern, with uniform, small elongated cells [3,7,10]. If melanin-containing cells are interspersed with DFSP cells, an uncommon variant of DFSP known as a Bednar tumor, should be considered [11]. Clinically, genetic testing is not commonly utilized, as histological appearance and immunohistochemical staining are typically sufficient. Nonetheless if staining is equivocal, DFSP has a distinguishable chromosomal translocation, t(17:22) (q22,q13), which leads to the formation of supernumerary ring chromosomes [12]. Many fusion genes are involved, with COL1A1 cited as the most common [13]. Due to its rarity, no standard treatment protocol exists, with reports of surgery, chemotherapy, and radiotherapy alone or in combination for varying stages of DFSP [14,15].

This case presents a number of aspects that defy common characteristics of DFSP, which highlights the difficulty in clinical diagnosis. DFSP is commonly discovered deep to the dermis, with associated telangiectasia and skin discoloration [5]. The case lesion, however, was subcutaneous and lacked overt dermatological manifestations other than the presence of a mass. The atypical presentation may be due to the immunocompromised status of the patient or scar tissue development from the previous excision, as both circumstances have been shown to complicate and delay DFSP diagnosis [15]. Induration is also a prominent sign of disease progression, but can be obscured by scar tissue and thus delay disease recognition [16]. DFSP is known to have a recurrence rate of approximately three years from incomplete excision and resection, in which the initial lipoma may have been misdiagnosed or masked an underlying DFSP [15]. As the patient presented within the recurrence window, it is important for the previous lipoma to be reexamined for possible error in diagnosis and consideration of early DFSP transformation [17].

The differential diagnosis for DFSP can be varied due to its non-specific physical manifestation. Surgical pathology considerations can include dermal and deep fibrous histiocytoma, myxoid nerve sheath tumor, myxoid liposarcoma, plexiform fibrohistiocytic tumor, desmoplastic tumor, and adult fibrosarcoma among others [18]. Immunohistochemistry is the gold standard for definitive diagnosis as CD34+ testing demonstrates high specificity and sensitivity for DFSP. Conversely, DFSP stains negatively for factor XIIIa, keratins, and S100c, all of which are hallmarks of other soft tissue tumors considered in the differential [19]. Robust follow-up and care continuity are required to prevent sequelae and recurrence, with multidisciplinary management between primary care, surgery, and interventional radiology playing key roles in optimizing care [20].

## 4 Conclusion

DFSP is a rare tumor of the skin and subcutaneous tissue. Though it commonly presents with induration, skin discoloration, and telangiectasias, it is important to keep DFSP as a differential in new masses found on previous surgical sites, especially if the previous mass had a possibility of recurrence.

## Declaration of Competing Interest

None.

## Sources of funding

None.

## Ethical approval

As a case report, this article does not meet DHHS definition of research and thus does not require review by Touro College of Osteopathic Medicine’s IRB and is exempt from this process. Efforts were made by all authors to ensure compliance with HIPAA requirements

## Consent

Written informed consent was obtained from the patient for publication of this case report and accompanying images. A copy of the written consent is available for review by the Editor-in-Chief of this journal on request.

## Registration of research studies

Name of the registry: N/A.

Unique identifying number or registration ID: N/A.

Hyperlink to your specific registration (must be publicly accessible and will be checked): N/A.

## Guarantor

Justin Chin.

## Provenance and peer review

Not commissioned, externally peer-reviewed.

## CRediT authorship contribution statement

**YaQun Zhou (Arlene):** Conceptualization, Investigation, Writing - original draft, Writing - review & editing. **Justin Chin:** Conceptualization, Methodology, Writing - original draft, Writing - review & editing. **Millard D. Strutin:** Investigation, Resources, Writing - original draft, Writing - review & editing, Supervision. **Christine M. Lomiguen:** Conceptualization, Writing - original draft, Writing - review & editing, Project administration.
